# The routes of infection spread in central skull**-**base osteomyelitis and the diagnostic role of CT and MRI scans

**DOI:** 10.1186/s12880-019-0331-7

**Published:** 2019-08-01

**Authors:** J. Mejzlik, M. Cerny, L. Zeinerova, J. Dedkova, J. Kopriva, K. Zadrobilek, J. Adamkov, V. Chrobok, V. Pellantova

**Affiliations:** 1 Department of Otorhinolaryngology and Head and Neck Surgery, Faculty of Medicine in Hradec Kralove, University Hospital Hradec Kralove, Charles University in Prague, Sokolska 581, Hradec Kralove, 500 05 Hradec Kralove, Czech Republic; 2 Department of Radiology, Faculty of Medicine in Hradec Kralove, University Hospital Hradec Kralove, Charles University in Prague, Hradec Kralove, Czech Republic; 3 Department of Neurosurgery, Faculty of Medicine in Hradec Kralove, University Hospital Hradec Kralove, Charles University in Prague, Hradec Kralove, Czech Republic; 4 Department of Infectious Diseases, Faculty of Medicine in Hradec Kralove, University Hospital Hradec Kralove, Charles University in Prague, Hradec Kralove, Czech Republic

**Keywords:** Central skull-base osteomyelitis, Imaging, Treatment, Cranial nerve palsy, computed tomography

## Abstract

**Background:**

Central skull-base osteomyelitis (CSBO) represents a life-threatening complication of external ear canal infection. Computed tomography (CT) and magnetic resonance imaging (MRI) play key roles in assessment of CSBO progression.

**Methods:**

Twelve patients with CSBO were included in a retrospective clinical study. In total, 62 scans (30 CTs and 32 MRIs) were performed to evaluate the extent of inflammatory changes. The scans were read independently by two radiologists specialised in imaging of the head and neck. The regions under the skull base were specified using the online Anatomy Atlas of the skull base. To clarify the timeline, the time period was divided into four parts, and inflammatory changes in the skull-base regions were tracked. Data were statistically analysed.

**Results:**

In early stages of the disease, CT scan detects inflammatory changes closely related to the stylomastoid foramen and medially to the posterior belly of the digastric muscle, changes which have been proved to be crucial for the diagnosis of CSBO. Later the infection spreads to the contralateral side causing demineralisation of the bones.

**Conclusion:**

Imaging methods play a crucial role not only in establishing the diagnosis, but also in anticipating the direction of infection spread underneath the skull base.

## Background

Malignant otitis externa usually begins as an external auditory canal skin infection followed by osteomyelitis of the temporal bone. Spread to the skull base occurs through the tympanomastoid suture to affect the stylomastoid and jugular foramina. Similarly, central skull-base osteomyelitis (CSBO) represents a rare but life-threatening complication, involving the temporal bone pyramid, clivus, sphenoid bone wings, and adjacent soft tissues beneath the central skull base. Typically, the CSBO infection begins in the external ear canal, but spread of infection from paranasal sinuses has also been reported [1]. CSBO usually develops slowly as otitis externa lasting for weeks or months. The infection spreads under the periosteum of the tympanic bone, crosses the petrotympanic and tympanomastoid sutures, and continues towards the temporal bone apex and clivus, and also reaches the bony structures on the contralateral side. The inflammatory changes can be traced not only within the bone but also in the soft tissues below the skull base. Manifestation of cranial nerve palsy in patients with otitis externa leads to suspicion of CSBO. The group most affected are elderly diabetics with recurrent otitis externa [2]. The most common infectious agents include *Pseudomonas aeruginosa*, *Staphylococcus* species, or fungi [1, 3]. The presence of symptoms such as severe otalgia, discharge and cranial nerve palsy (VI, VII, VIII, IX, X, XI, XII) are crucial for establishing the diagnosis of CSBO [2], as laboratory parameters are usually inconclusive and the results of both biopsy and microbial culture are frequently negative.

CT is used abundantly to determine the extent of temporal bone lesions. For example, in chronic otitis media with cholesteatoma the preoperative classification is based on CT examination [4]. Magnetic resonance imaging (MRI) is essential for determining soft tissue involvement, and in the area of the external auditory canal, not only the size of the tumour but also the type of malignancy can be judged on the basis of diffusion-weighted MRI [5]. In CSBO, computed tomography (CT) demonstrates incipient bone destruction, but demineralization of the bones is evident in CT as a late sign of inflammation. Magnetic resonance imaging (MRI) reveals alterations of the clivus on T1-weighted unenhanced images. ^67^Gallium scintigraphy interestingly reveals higher metabolic activity in the temporal bone pyramid and the clivus. To monitor the spread of inflammation in the cranial base, repeat CT or MRI examinations should be performed. In the area of the cranial base, near the dentition, a number of artefacts can degrade both CT and MRI images. Radiation exposure can be reduced by low-dose CT and post-processing data can minimize artefacts [6]. The Curve-Like Structure Extraction method may help to track linear structures such as fracture lines or vessels [7]. The objective of this research is to highlight the role of CT in detecting the moment of infection spread from the temporal bone to the central skull base. A CT scan, even non-contrast enhanced, in the early stages of the disease can identify the soft tissue changes before the osteolysis becomes obvious.

## Methods

Eleven male and one female patients with CSBO were identified and assessed retrospectively in the Department of Otorhinolaryngology and Head and Neck Surgery, University Hospital Hradec Kralove during the period 2012–2017. The data were collected from medical records. The average age of patients was 78.0 years (range 70–85 years), average weight 84.1 kg (range 57-101 kg), and average body mass index (BMI) 29.1 (range 21.1–38.1). All the patients had various comorbidities including diabetes mellitus (DM) 6 (50.0%), ischemic heart disease (IHD) 4 (33.3%), renal failure (RF) 4 (33.3%), chronic pulmonary disease (PD) 7 (58.3%), and others 5 (41.7%).

The disease progressed over time in all cases, and the patients were admitted to the hospital at different stages, with an average hospitalisation time of 20 days (range 4–40 days). The inclusion criteria were: otitis externa not responsive to conservative outpatient treatment; characteristic clinical findings such as severe otalgia, ear discharge, one or more cranial nerve palsies (VI, VII, VIII, IX, X, XI, XII); osteolysis of the clivus on CT or alteration of the clivus on MRI.

In all cases one or more biopsies were obtained from inflamed regions: 3 (25%) biopsies were from mastoid cells, 9 (75%) from the retropharyngeal space trans-nasally, and 1 (8.3%) orbital.

Malignancy, mycobacterial infection and granulomatosis with polyangiitis were always excluded by laboratory findings, imaging methods and biopsy.

Microbiological cultures were taken both prior to and after antibiotic therapy. CT investigation of the temporal bones was performed as a first imaging method, followed by MRI. Both CT and MRI scans were reviewed retrospectively. Altogether 62 scans were performed, of which 30 were CTs and 32 MRIs. The scans were read independently by two radiologists specialised in imaging of the head and neck. High Resolution Computed Tomography (HRCT) was the preferred imaging method to exclude osteomyelitis of the temporal bone. Once the diagnosis of CSBO was confirmed, the disease was followed up by non-contrast enhanced MRI; hence the difference in the numbers of CTs and MRIs.

The regions under the skull base were specified according to the terminology from the online Anatomy Atlas of the skull base: external_meatus, clivus, longus_capitis_m, rectus_capitis_m, ICA, petrous_apex, mastoid, foramen_lacerum, Eustachian_tube, medial_pterygoid_m., lateral_pterygoid_m, tensor_velipalatini_m, levator_veli_palatini_m, torus_tubarius, digastric_m, bulbus_jugularis, styloid_process, foramen stylomastoideum, condylus_occipitalis [8].

The patients were followed up for different periods of time. To simplify the timeline, the follow-up period was structured into four parts: day 0, days 5–75, days 93–177, and days 209–477.

Inflammation levels were tracked using erythrocyte sedimentation rate (ESR), C-reactive protein (CRP), and white blood cell count (WBC).

The data from all the laboratory results of the patients were gathered, and missing data (10%) were ignored in the analysis. Statistical analysis was performed using the analytical software Statistica version 13.3. Descriptive data are presented as a number (percent in the file) or the median (range). Pearson Chi-square statistics were used to evaluate the statistical significance (p) of DM, IHD, RF, PD, bacterial branches, stylomastoid region involvement, and time of hospital stay after diagnosis of CSBO. Regression analysis was performed to determine the impact of individual factors on the observed variables. Data are presented as odds ratio (OR), (95% confidence interval (CI)), and p.

## Results

Microbiological cultures revealed one or more bacterial strains including *Pseudomonas aeruginosa* in 9 cases (75%), *Staphylococcus aureus* in 8 cases (66.7%), *Candida albicans* in 2 cases (16.7%), *Citrobacter* in 1 case (8.3%), *Candida parapsilosis* in 1 case (8.3%), *Malassezia furfur* in 1 case (8.3%), *Staphylococcus* coagulase-negative in 1 case (8.3%), *Stenotrophomonas maltophila* in 1 case (8.3%), and *Klebsiella pneumoniae* in one case (8.3%). Biopsies showed acute or sub-acute inflammatory granulation tissue covered by squamous or respiratory epithelium in all cases.

Palsy of one or more cranial nerves developed in all cases (Table 1).

**Table 1:** List of cranial nerves palsies. (n. VI. – n. XII.) which were diagnosed during the course of the disease. Abducens nerve (VI.) palsy is manifested by a disturbance of lateral eye bulb movement; facial nerve (VII.) palsy by movement disorder of half of the face; vestibulocochlear nerve (n. VIII.) palsy by sensorineural hearing loss and disequilibrium; glossopharyngeal nerve (IX.) palsy by disorder of soft palate mobility; vagus nerve (X.) palsy by unilateral vocal cord paresis; accessory nerve (XI.) palsy by inability to raise the arm above the horizontal; hypoglossal nerve (XII.) palsy by tongue movement disorders. Most patients had more than one cranial nerve palsy

Cranial nerve palsy	No.	% *n* = 12
n.VIII.	7	58.3
n.VII.	6	50.0
n.IX.	3	25.0
n.X.	3	25.0
n.XII.	3	25.0
n.VI.	1	8.3
n.XI.	1	8.3

At the time of admission the average levels of inflammation markers were: ESR 60.5 mm/1 h (range 1.3–219.5)/ 80.2 mm/2 h (range 24–149); CRP 33.1 mg/l (range 1.3–219.5); WBC 8.3 × 10^9^/l (range 2.9–16.6).

**Fig. 1:**
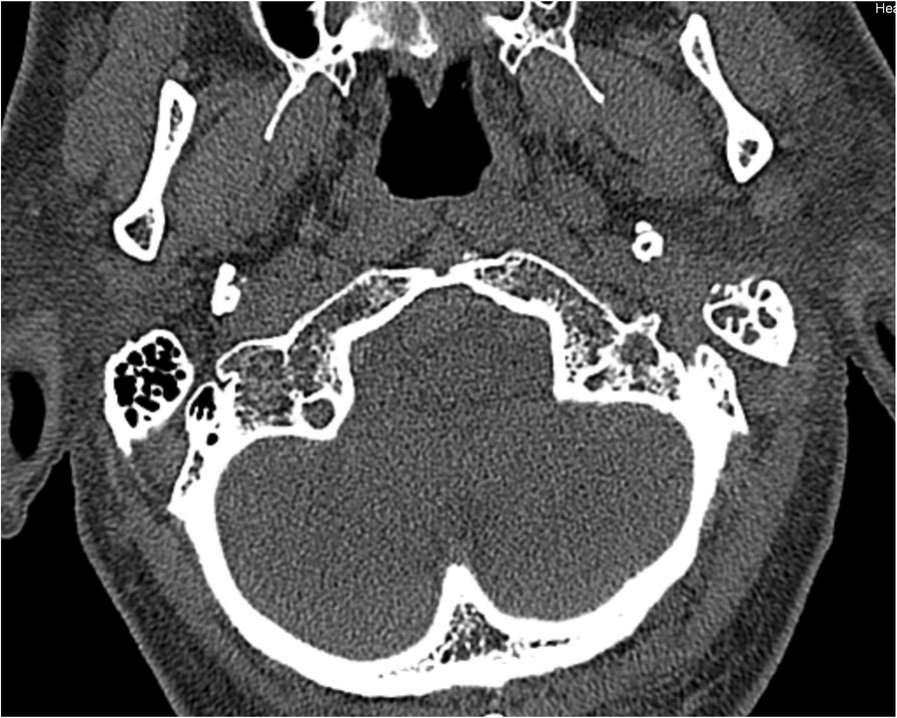
Day 0. Non-contrast enhanced CT in axial plane, section thickness 1 mm. The fatty tissues adjacent to the stylomastoid foramen are infiltrated, and cortical bone within the mastoid process is discretely eroded. The mastoid cells are filled with oedematous mucosa and soft tissue

**Fig. 2:**
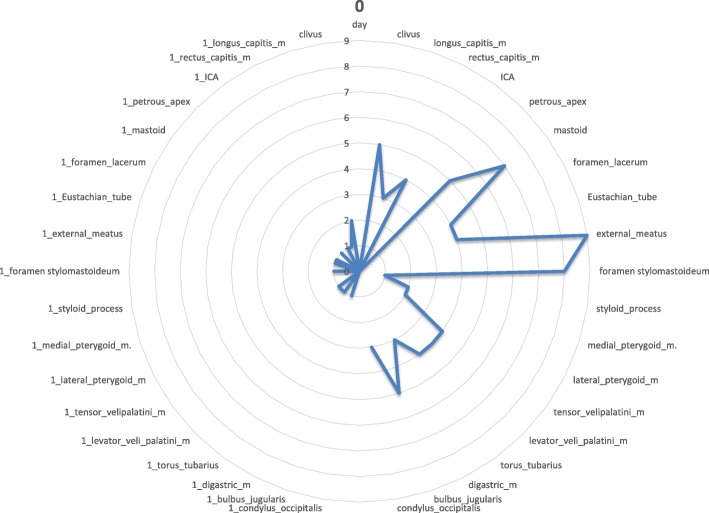
Day 0 pattern of affected regions on the right side and on the contralateral left side. The outer ear canal and stylomastoid foramen are significantly affected

**Fig. 3:**
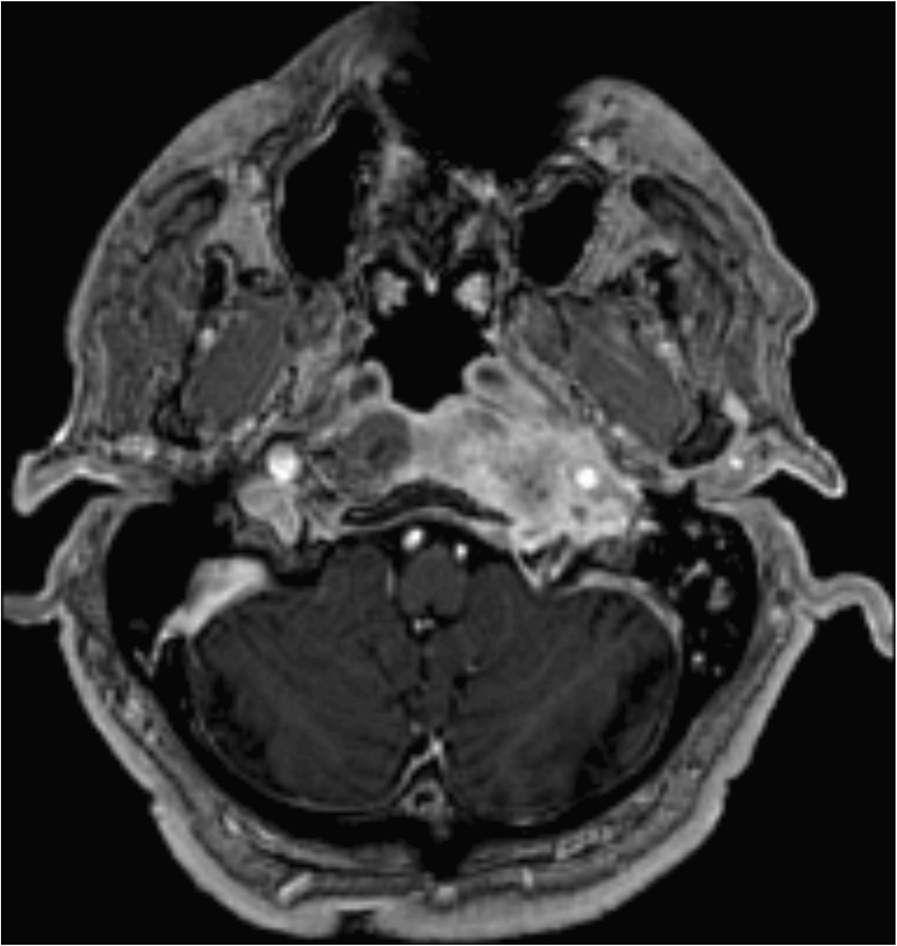
Day 5–75. Gadolinium-enhanced MRI, axial plane, T1-weighted, with fat suppression. Signal is homogeneously increased in the Eustachian tube and longus colli muscle. Cortical bone within the skull base is eroded, specifically the basal portion of the occipital bone ventrodorsally

**Fig. 4:**
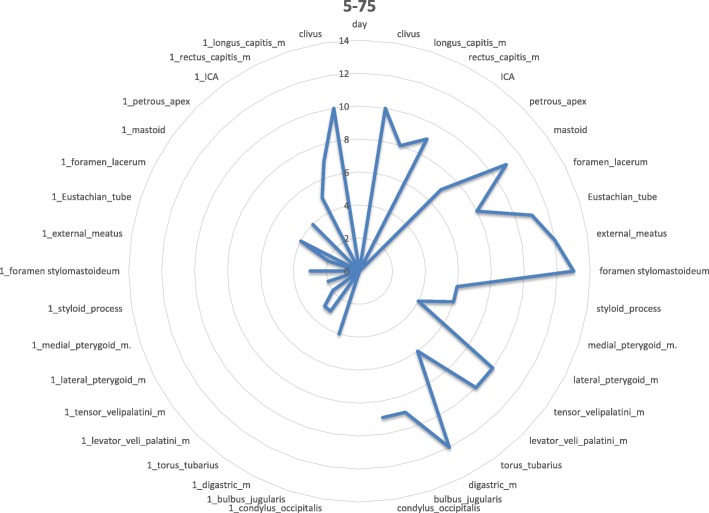
Day 5–75: the inflammation spreads to further regions on the affected side

**Fig. 5:**
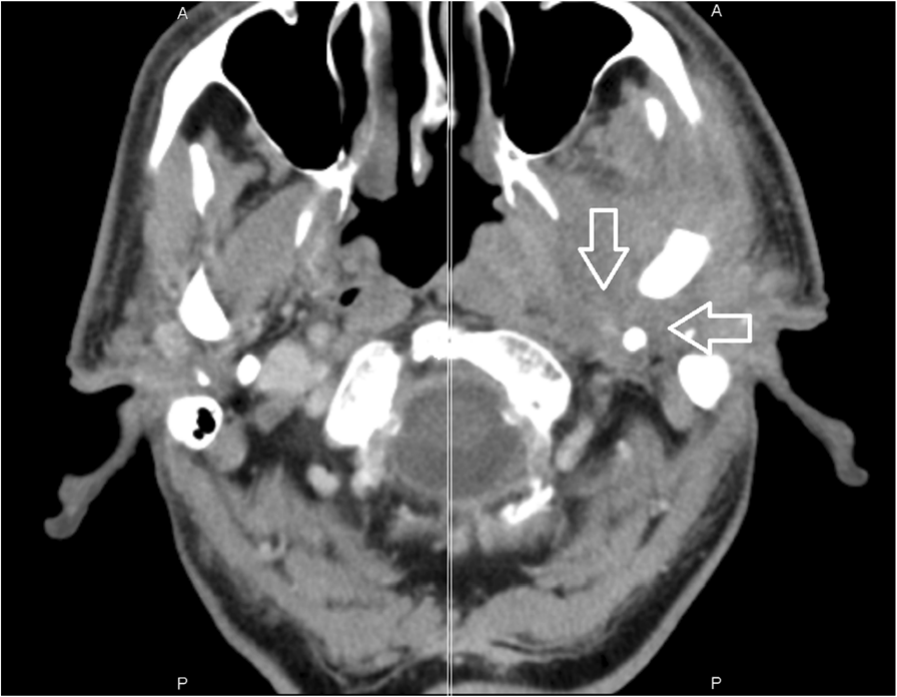
Contrast-enhanced axial CT image of temporal and occipital bones. Oedematous changes in soft tissues adjacent to the stylomastoid foramen on the left side represent the first sign of osteomyelitis spread beyond the temporal bone (arrows)

**Fig. 6:**
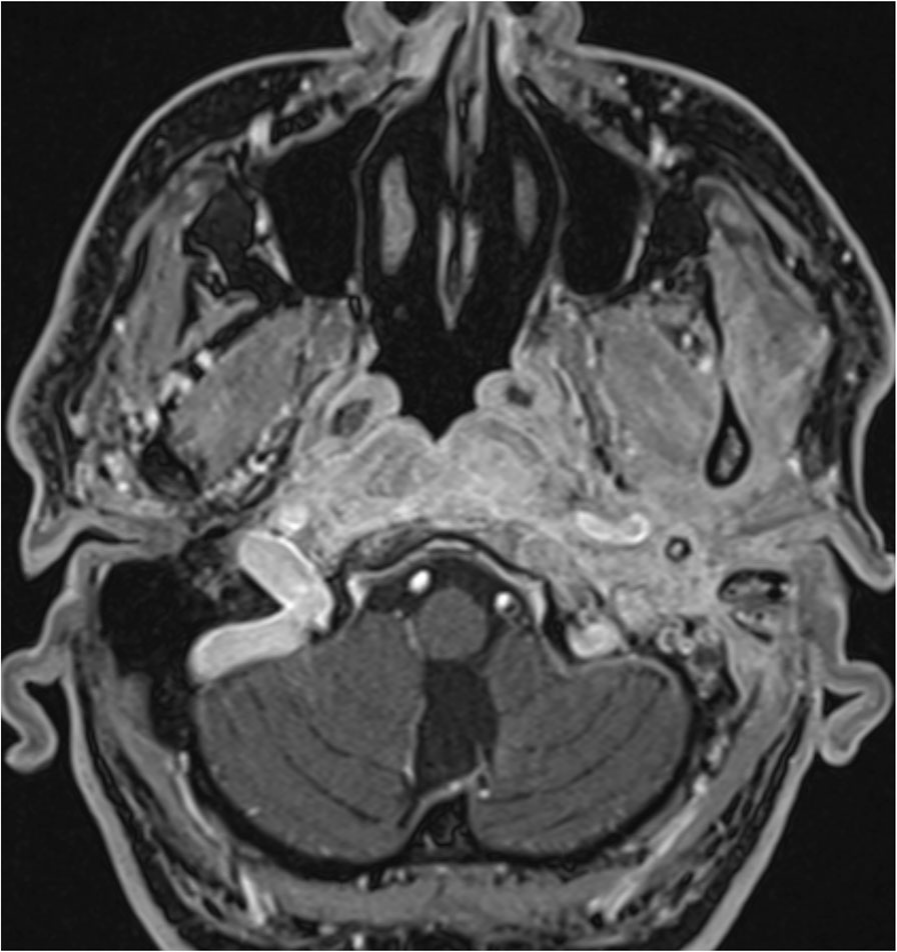
Day 93–177. Gadolinium-enhanced MRI, axial plane, T1-weighted, with fat suppression. The signal increases homogeneously on the left side in retrostyloid and pre-styloid regions, longus colli muscle, and Eustachian tubes on both sides. The signal mildly and homogeneously increases in the temporomandibular joint, lateral pterygoid, and masseter muscles on the left side. Bone marrow shows signs of infiltration in the basal part of the occipital bone

**Fig. 7:**
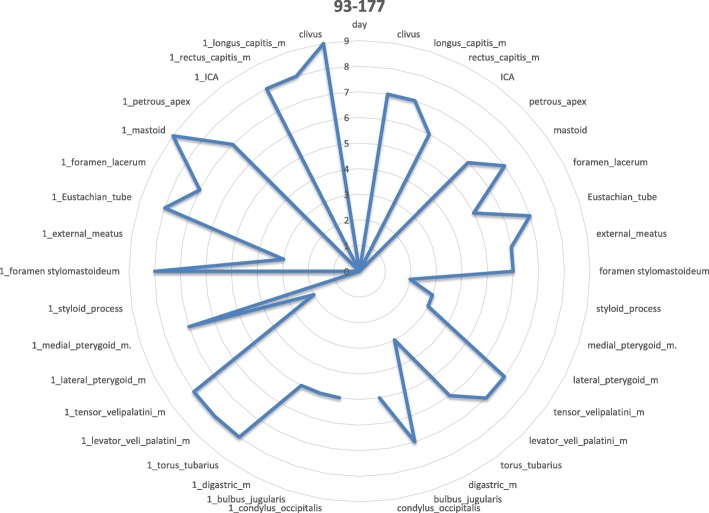
Day 93–177: massive spread of infection to the contralateral side (to the left)

The involvement of selected regions was assessed (Table 2). In all 12 cases, clinical infection started in the external auditory canal. By the time the CSBO diagnosis was made, changes on imaging were present in only 9 patients. In the early stages of the disease, CT scans showed involvement of the soft tissues surrounding the external ear canal without evidence of bony lesions. The individual structure involvement is depicted in Figs. 1 and 2 provides details of tissue involvement on primary imaging evaluation. As the disease progressed, CT scans detected inflammatory changes in fat tissue closely related to the stylomastoid foramen medially to the posterior belly of the digastric muscle (Figs. 3 and 4). Once reviewed, this latter finding was found crucial for the diagnosis of CSBO (Fig. 5). Only in the later stages of the disease did non-enhanced T1-weighted images display destruction of the tympanic and temporal bones, osteolytic changes, and alterations of the clivus, as well as enhancement of the soft tissues beneath the skull base (Figs. 6 and 7).

**Table 2:** Overview of affected regions on the affected and contralateral sides during the time period: day 0 was the day of establishing the diagnosis, days 5–75 represent early stage, days 73–177 represent advanced stage, and days 209–477 represent follow-up. Results from the affected and contralateral sides are in separate halves of the Table

	Affected				Contralateral			
Days	0	5–75	93–177	209–477	0	5–75	93–177	209–477
External_meatus	9	12	6	2	0	0	3	3
Clivus	5	10	7	7	2	10	9	7
Longus_capitis_m	3	8	7	6	1	7	8	5
Rectus_capitis_m	4	9	6	6	1	5	8	5
ICA	0	0	0	0	0	0	0	0
Petrous_apex	5	7	6	7	1	4	7	5
Mastoid	7	11	7	4	0	0	9	5
Foramen_lacerum	4	8	5	7	1	4	7	5
Eustachian_tube	4	11	7	6	1	2	8	5
Medial_pterygoid_m.	2	6	3	0	0	2	7	3
Lateral_pterygoid_m	2	4	3	0	0	0	2	3
Tensor_velipalatini_m	4	10	7	6	1	2	8	5
Levator_veli_palatini_m	4	10	7	6	1	3	8	5
Torus_tubarius	4	6	6	6	1	3	8	5
Digastric_m	3	12	3	0	0	0	5	3
Bulbus_jugularis	5	9	7	4	1	4	5	2
Styloid_process	1	6	2	0	0	0	0	0
Foramen stylomastoideum	8	13	6	0	1	3	8	3
Condylus_occipitalis	3	9	5	2	0	0	5	0

In all 12 patients, CT scans confirmed the presence of fluid in the mastoid cells. However, this is a non-specific finding, and can be seen even in individuals without active inflammatory processes. In one patient, the infection also involved the temporomandibular joint and the upper eyelid on the affected side.

**Fig. 8:**
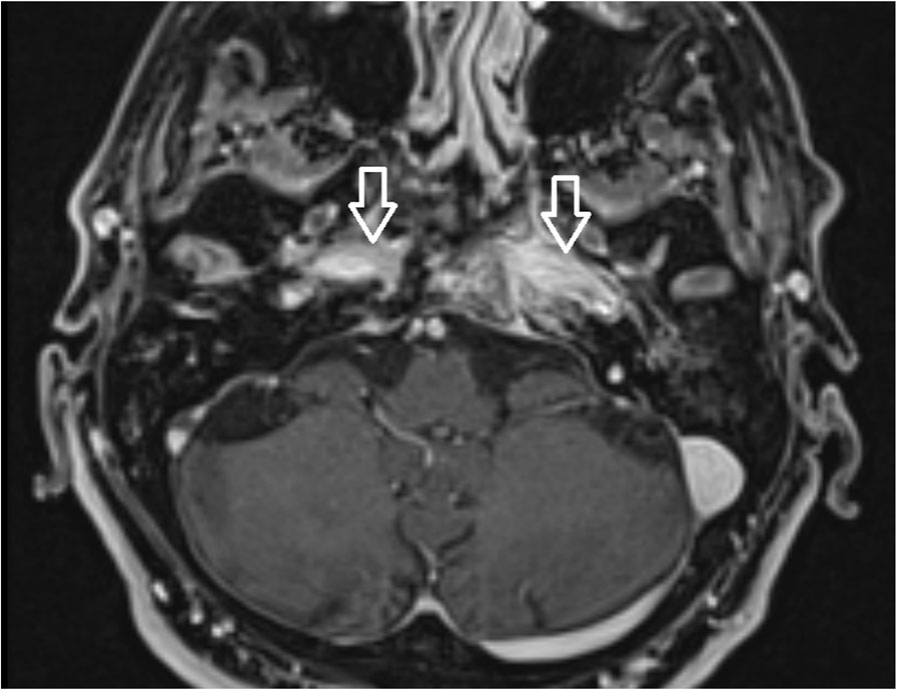
Gadolinium-enhanced axial T1-weighted spin-echo image with fat saturation. In advanced stages of an infection, a butterfly-shaped soft tissue enhancement was described underneath both petrosal bones and the clivus (arrow)

**Fig. 9:**
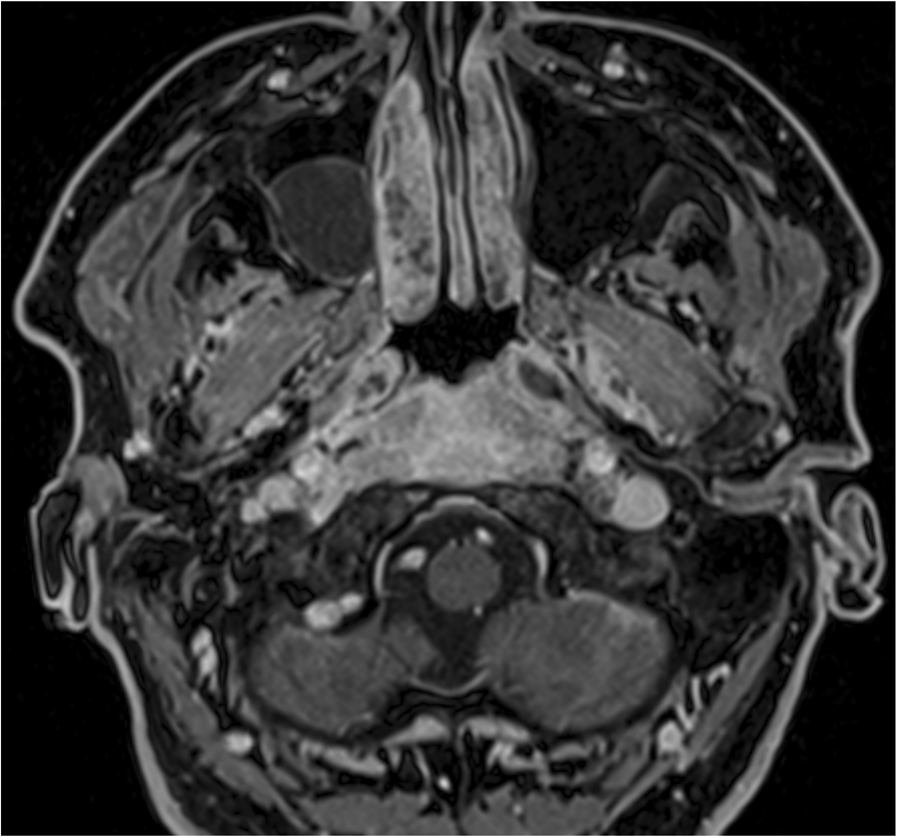
Day 209–477. Gadolinium-enhanced MRI, axial plane, T1-weighted, with fat suppression. Mild homogeneous signal increase persists in the pharyngobasilar fascia and longus colli muscles on both sides. A cyst in the right maxillary sinus represents a secondary finding

**Fig. 10:**
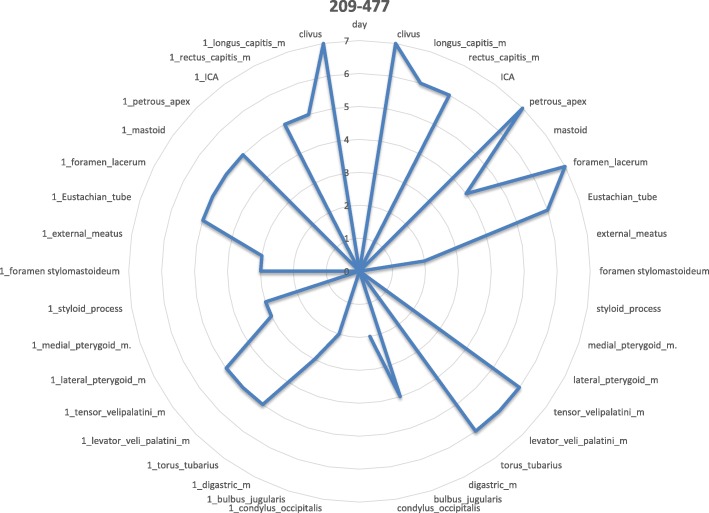
Day 209–477: slow infection improvement in all regions on affected and contralateral sides

Additionally, MRI investigations in the early stage of the disease again illustrated fluid in the mastoid cells and oedema of the soft tissues adjacent to the external ear canal. Soft tissue enhancement was visible on axial gadolinium-enhanced T1-weighted imaging, originating from the digastric muscle insertion and continuing to the insertions of the musculus longus capitis and musculus rectus capitis anterior (Fig. 8). The pharyngobasilar fascia was in close contact with the pharyngeal process of the ossis occipitalis in the middle line, and represented the frontal border of the infection. Contralateral spread beneath the clivus in the retropharyngeal space was consistently noted. In advanced stages of infection the butterfly-shaped soft tissue enhancement was located underneath both petrosal bones and the clivus (Figs. 6 and 7). Progression of infection from the temporal bone to the other skull base bones was not noticed, except for progression through the fissura sphenooccipitalis towards the occipital bone (Fig. 8). When the treatment proved to be successful, gradual improvement of inflammatory changes in the individual regions was observed (Figs. 9 and 10).

### Therapy

All patients were treated with oral antibiotics as outpatients prior to admission to the hospital. As the diagnosis of CSBO was confirmed, oral or intravenous systemic antibiotic treatment was started according to the specific microbial sensitivity and findings (Table 3).

**Table 3:** Antibiotics used for the treatment of the CSBO. The antibiotic choice reflects the two most frequently detected bacteria, *Pseudomonas aeruginosa* 9 (75%) and *Staphylococcus aureus* 8 (66.7%). Both were detected using microbiological culture and tested for antibiotic resistance

Antibiotics	No.	% n = 12
Ciprofloxacin	11	91.7
Ceftazidime	9	75.0
Amoxicillin/clavulanic_acid	9	75.0
Gentamycin	4	33.3
Clindamycin	4	33.3
Meropenem	4	33.3
Clarithromycin	3	25.0
Dalacin	3	25.0
Ofloxacin	3	25.0
Piperacillin	3	25.0
Cefuroxime_axetil	2	16.7
Rifampicin	2	16.7
Oxacylinum	2	16.7
Amikacin	2	16.7
Vancomycin	1	8.3
Linezolid	1	8.3
Co-trimoxazole	1	8.3
Clarithromycin	1	8.3

Mastoidectomy was indicated for facial nerve paralysis progression, headaches, persisting ear discharge, or for osteolytic changes of the temporal bone indicated on CT. Histology revealed mild nonspecific inflammatory changes in all cases. In one case, a thyroplasty type I was performed for hoarseness and aspirations caused by cranial nerve X palsy. In 10 cases, local control of infection was established, cranial nerve palsies disappeared, and headaches improved, although changes in soft tissues persisted on MRI. Despite intensive antibiotic therapy, 2 patients died in the regional hospitals as a result of the disease.

### Statistical analysis

Analysis was focused on variables that would be early predictors of CSBO development. As this is a retrospective study, the data were not taken consistently in the timeline of the disease. Pearson Chi-square test was found to be suitable for testing the statistical significance (p) of variables. From the range of all possible variables that were tested, comorbidities: DM, IHD, RF, PD, others; bacteria: *Pseudomonas aeruginosa*, *Staphylococcus aureus*; and stylomastoid involvement and duration of hospital stay were found to be of interest. However, only stylomastoid involvement achieved statistical significance *p* = 0.021 (Table 4).

**Table 4:** Pearson Chi-square statistics were used to find a factor that would predict the occurrence of CSBO. The list contains factors with major statistical importance: (DM) diabetes mellitus, (IHD) ischemic heart disease, (RF) renal failure, (PD) pulmonary disease. Infection in the stylomastoid region on imaging methods was found to be the only statistically significant factor

	Chi-Square	p.
DM	0.000	1.000
IHD	1.33	0.248
RF	1.33	0.248
PD	0.33	0.564
Others	0.33	0.564
*Pseudomonas aeruginosa*	4.5	0.105
*Staphylococcus aureus*	1.33	0.248
Stylomastoid involvement	**5.33**	**0.021**
Hospital stay duration	0.83	1.000

In the knowledge that this was statistically significant, effort was focused on detection of parameters related to stylomastoid involvement. Among the variables tested, the ESR_mm_1h: OR 1.041, 95%CI 1.010–1.073, p 0.027; and ESR_mm_2h: OR 0.980, 95%CI 0.9511.000, p 0.039 were found to be statistically significant, albeit the OR is relatively low (Table 5).

**Table 5:** Regression analysis of the inflammatory markers associated with: 1) dependent variable: stylomastoid involvement; 2) independent variables: ESR after 1 and 2 h were found to be statistically significant but OR remains low

	OR	95% CI		p.
CRP	1.010	0.990	1.020	0.281
WBC_x10E9_l	1.020	0.951	1.083	0.569
ESR_mm_1h	**1.041**	**1.010**	**1.073**	**0.027**
ESR_mm_2h	**0.980**	**0.951**	**1.000**	**0.039**

The duration of hospital stay was also tested as a marker of seriousness of the disease, but was not found significant.

## Discussion

Infections of the external auditory canal are not always easy to cure, particularly if the infection spreads inside the tympanic, mastoid or petrous bones. The most advanced stage of the infection is CSBO. The treatment recommendations are usually based on case reports or case series reports [2]. Early diagnosis based on clinical symptoms and imaging is crucial for successful therapy. Unfortunately, early diagnosis is often missed as the patients are presented to the ENT department after weeks or months of problems [9], and this was the experience in our group. Once the greater part of the skull base has been affected, the diagnosis becomes easier to establish but the prognosis worsens. Thus the authors highlight the importance of early diagnosis that allows an early start to treatment.

### Imaging

Medical imaging techniques represent a valuable method for diagnostics and follow-up. HRCT is used to exclude bone involvement in malignant otitis externa. In our group, HRCT was always performed as the first imaging method.

Contrast-enhanced CT scans focus on soft tissue imaging beneath the skull base, gadolinium-enhanced MRI scans are excellent for tracking the involvement of the clivus, and ^67^Ga scintigraphy can evaluate skull base metabolic activity [10]. The MRI finding typically includes enhancement of the clivus with hypointensity in the bone marrow space on T1-weighted images, hyperintensity on T2-weighted images, and effacement of the parapharyngeal fat planes and soft tissues at the skull base [11]. Diffusion-weighted imaging can increase the prominence of the skull lesions and can be used to distinguish between malignant and benign lesions [12, 13]. To exclude malignancy, one or more biopsies were usually taken. Without exception, the CT or MRI scans were performed for neurological reasons to exclude stroke prior to ENT examination. Once the diagnosis had been established, the MRI was repeated bi-monthly.

**Fig. 11:**
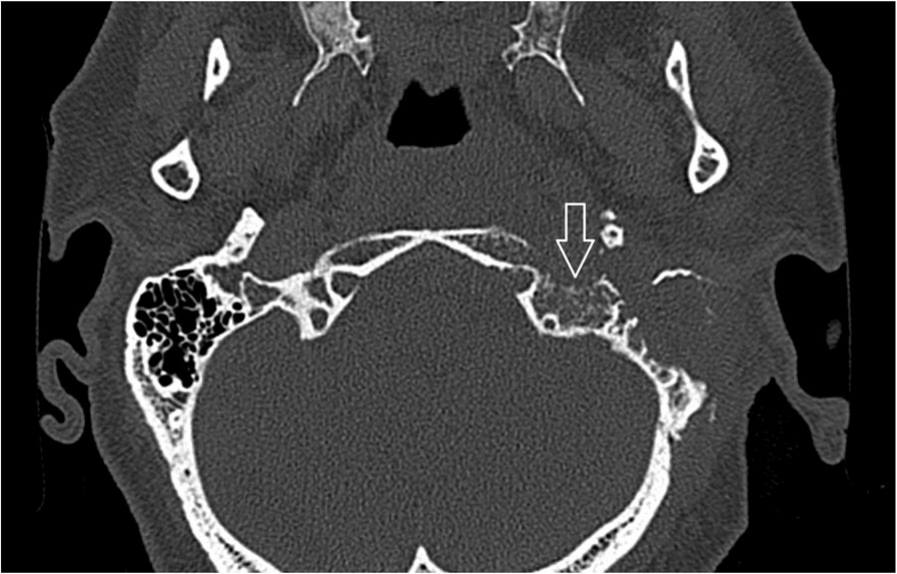
Patient No: 3, five months following mastoidectomy and antibiotic treatment: amoxicillin/clavulanic acid, gentamycin, clarithromycin and dalacin. Non-enhanced CT image (bone window) axial scan cross section of temporal and occipital bones. Osteolysis of the compact bone in the left lateral skull base represents a late sign of osteomyelitis (arrow). Minor changes of spongy bone and major changes of compact bone highlight spread of infection under the periosteum, but represents a late sign of the infection

Sreepada et al. consider the tympanomastoid suture crucial for spread of infection [14]. The swelling of soft tissues adjacent to the tympanomastoid and petrotympanic sutures and stylomastoid foramen was visible in all cases on non-contrast CT scans. Therefore the authors consider it as a warning sign suggestive of further progression of skull base infection, similarly as reported in malignant otitis externa [15]. Sreepada also recommends CT follow-up for CSBO even though the osteolytic changes of the compact bone on CT represents a rather late sign, as 30% of bone must be demineralized to appear eroded on CT scans (Fig. 11). If soft tissue changes were visible on non-contrast enhanced CT scans, the suspicion of inflammation was raised. MRI is considered essential for CSBO diagnosis [16]. Venous channels and fascial planes facilitate the spread along the dural venous sinuses. Two experienced radiologists were asked to trace the infection spread on CT and MRI scans. After acquisition of the data, the pathways can be traced even by post-processing imaging methods [7]. Hence, most active disease was found in the compact bone along the middle and posterior cranial fossa surfaces with extension to the petrous apex [14]. In patient No 2 the infection also involved the temporomandibular joint and parotid gland and the upper eyelid on the affected side. This was due to an anatomical variant, persistent foramen of Huschke, which allowed infection spread from the external auditory canal to the temporomandibular joint [17]. In this region, advanced diffusion imaging MRI can be used [18]. The rudimentary foramen was also observed in one case and facilitated infection spread towards the temporomandibular joint and surrounding tissues.

**Fig. 12:**
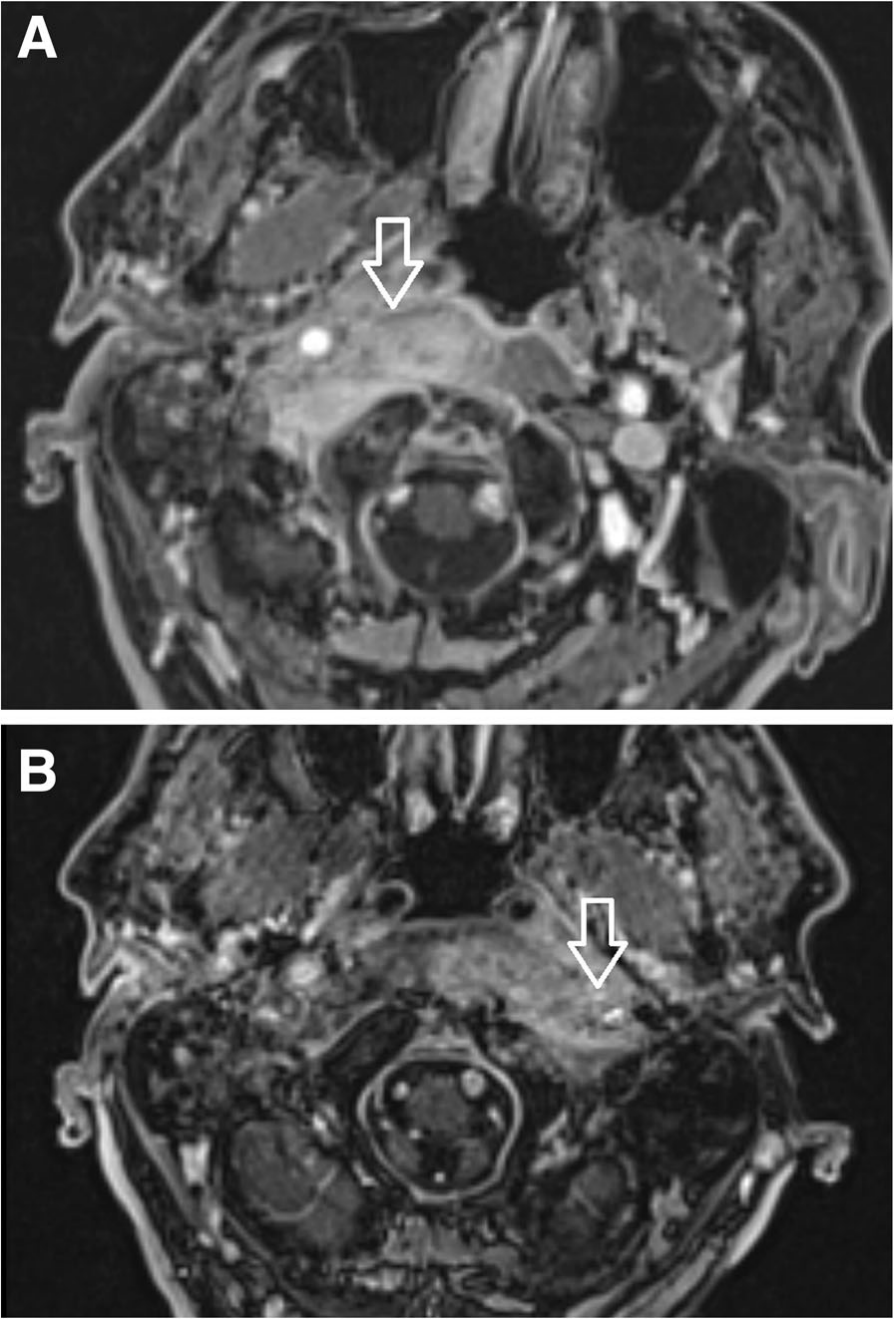
**a** Patient 1, 2 months after the first clinical symptoms: MRI of the skull base. Contrast-enhanced axial T1-weighted spin-echo image with fat saturation. Inflammatory changes of the right side soft tissues beneath the skull base, musculus longus capitis and musculus rectus capitis anterior (arrow). The swelling extends to the midline; the infection originates from the right external auditory canal. **b** Patient No. 1, 4 months following the first clinical symptoms: MRI of the skull base of the same patient. Contrast-enhanced axial T1-weighted spin-echo image with fat saturation. After antibiotic treatment: ceftazidime, ciprofloxacin, clindamycin, Oxacyllin. Inflammatory changes of the soft tissues below the skull base progressed to the left side (arrow), musculus longus capitis and musculus rectus capitis anterior. The swelling affects the retropharyngeal and retrostyloid part of the parapharyngeal space in the midline and progresses to the left side

MRI represents an excellent technique for soft tissue imaging. Fascial spaces, periosteum, muscular insertions and bone marrow are visible on post-contrast T1 weighted scans. Sutures between skull bones represent a temporary barrier for osteomyelitis extension to adjacent bones. The periosteum within the skull base adheres more tightly to musculus rectus capitis anterior and musculus longus capitis insertions. Additionally, the periosteum, muscular tendons and fascia beneath the skull base are the boundaries for infection spread under the skull base. Those margins are the reasons for soft tissue enhancement in post-contrast T1 weighted imaging, which is typically a butterfly shaped zone of increased signal intensity between the spine and pharyngobasilar fascia. An interesting finding of infection spread was noticed on repeated MRI scans. The infection crossed the midline and spread through the retropharyngeal and retrostyloid parts of the parapharyngeal space into the contralateral temporal bone (Fig. 12. a, b). Origination of the pharyngobasilar fascia in the skull base represents a relatively steady border for infection spread. However, spaces anteriorly and laterally from the mentioned fascia are opened towards the sphenoidal bones and orbits. Crucial advantages in MRI scanning were reported by Ozgen et al. [16]. MRI scans are recommended to distinguish between malignant and benign lesions, and Ginat et al. recommend diffusion weighted imaging (DWI) [12].

### Diagnostics

Malignancy should be excluded in the first instance [2]. However, this is not possible without biopsy and histological examination. All cases were confirmed histologically. The presence of acute or subacute inflammatory changes had no impact on further management, but rather therapeutic response was guided by inflammation markers. Our small number of patients does not permit deeper statistical analysis; however, the largest reported meta-analysis [2] included 42 patients in 68 articles over a period of more than 60 years. It was proved that only the ESR correlates with the disease activity [2, 19]. Thus we conclude that our file of 12 patient merits consideration. The literature mostly recommends the use of MRI as the most valuable imaging method to determine the extent of infective changes of soft tissues below the skull base [1, 20]. Scintigraphy was not considered in our study, but new molecular tracers for assessment of bacterial infection have already been tested [21].

### Therapy

The bacterial strains found in our study were various, but it is well known that patients with CSBO frequently have been treated with a range of antibiotics prior to hospital admission, and cultures of biopsy material are very often sterile [10].

Both malignant otitis externa and CSBO should be treated by an ENT specialist [22]. Johnson and Batra systematically reviewed a series of 42 cases and recommended conservative management and long-term antibiotic treatment covering *Pseudomonas aeruginosa* or other infectious agents confirmed by cultivation [2, 19]. *Pseudomonas aeruginosa* infection should be suspected even if the microbial cultivation is negative [10], and hence the first choice of antibiotic treatment in our group of patients on admission was ciprofloxacin. Although in all cases the initial course of antibiotics was administered intravenously, an experimental intra-arterial application of antibiotic has also been reported [22]. Despite a number of papers unambiguously recommending conservative management of CSBO, some authors prefer a surgical approach associated with extensive treatment of affected bones [1]. We provided mastoidectomy in cases of deterioration in facial nerve palsy and the progression of osteolytic changes on CT. Two patients died due to renal and heart failure, exacerbated by long-lasting infection and even the side effects of the prolonged administration of antibiotics. We therefore conclude, in accordance with the literature, that comorbidities represent the main prognostic factor for survival [23].

## Conclusions

For the establishment of CSBO, CT and MRI findings proved to be crucial. Both surgical and conservative treatment of CSBO are dependent on CT and MRI scan findings. Before the inflammation starts to spread towards the clivus, the fat tissues adjacent to the styloid process becomes stranded on enhanced CT scans and the tissue changes are obvious even on non-enhanced CT scans. We consequently strongly recommend that CT or MRI imaging of the skull base becomes compulsory in elderly diabetics with otitis externa lasting more than 2 months.

## Abbreviations

BMI: Body mass index
CI: Confidence interval
CRP: C-reactive protein
CSBO: Central skull base osteomyelitis
CT: Computed tomography
DM: Diabetes mellitus
ESR: Erythrocyte sedimentation rate
HRCT: High resolution computed tomography
IHD: Ischemic heart disease
MRI: Magnetic resonance imaging
OR: Odds ratio
p: Statistical significance
PD: Pulmonary disease
RF: Renal failure
WBC: White blood cell count
